# Socio-ecological challenges and food security in the ‘salad bowl’ of Fiji, Sigatoka Valley

**DOI:** 10.1007/s10113-023-02059-6

**Published:** 2023-04-05

**Authors:** Kerrie Pickering, Tristan Pearce, Lui Manuel, Brendan Doran, Timothy F. Smith

**Affiliations:** 1 Sustainability Research Centre, University of the Sunshine Coast, 90 Sippy Downs Drive, Sippy Downs, QLD 4556 Australia; 2 Environmental Sustainability Research Centre, Brock University, St. Catharines, Canada; 3 Department of Geography, Earth, and Environmental Sciences, University of Northern British Columbia, 3333 University Way, Prince George, BC 42N 4Z9 Canada; 4 Department of Environment, Nadroga-Navosa Provincial Council, Talenavuruvuru, Lawaqa, P.O. Box 267, Sigatoka, Fiji; 5 SWEDESD, Department of Children and Women’s Health, Uppsala University, Uppsala, Sweden

**Keywords:** Adaptation, Fiji, Food security, Climate change, Resilience, Vulnerability

## Abstract

This article examines food security in the Sigatoka Valley, one of the most productive food regions in Fiji, in the context of recent socio-ecological challenges through a case study of Narewa village. Data were collected using semi-structured interviews (*n* = 25), a fixed question food insecurity experience survey (*n* = 25), and a free listing exercise about preferred and consumed foods (*n* = 24). Results revealed that while most households had access to sufficient food, the increased frequency and intensity of droughts, tropical cyclones, and flooding caused almost half to worry about meeting their future food needs. To date, a culture of sharing within the village has helped most households access food but this will likely be inadequate to meet future needs as climate change is projected to impact food production. Given that the foundation of food production in Narewa, like other villages in the valley, relies on the long-term viability of agricultural systems, better focus needs to be placed on the natural resources that form the backbone of these systems such as water availability, soil health, and slope stability and their resilience to anthropogenic and natural stressors. Efforts that focus on protecting and enhancing local ecosystems in light of expected future climate change, combined with greater attention on food storage and the use of resilient crops, and enhancing social cohesion and sharing networks are needed to avoid breaching tipping points in the food system.

## Introduction

Food security exists when people at all times can acquire safe, nutritional, and culturally acceptable foods in a manner that maintains human dignity (van Esterik 1999; Food and Agriculture Organization (FAO) 2002). Food insecurity occurs when food systems are stressed so that food is not accessible, available, and/or of sufficient quality. Food security in Pacific Island countries (PICs) varies by island geography and culture, ranging from inland and coastal communities in large islands with considerable natural assets to those that occupy small, low-lying atolls with little or no soil and limited fresh water resources. Historically, food security was sustained through agroecological biodiversity, the production of surpluses which enabled food preservation and storage to be practiced, and underpinned networks of exchange and mutual support that were particularly important during times of hardship, the use of resilient crops, and using ‘famine’ foods (Campbell 2015). A consequence of the political and economic changes experienced by PICs since the last half of the twentieth century, colonization, the spread of capitalism, introduction of new religions, migration, population increase, and urbanization have impacted traditional social structures and driven a transition from subsistence to market-based economies, which has in turn profoundly affected dietary intake and physical activity (Ulijaszek 2005). Many traditional food security practices have declined or disappeared, crop diversity has been reduced to make way for export crops, many traditional sharing networks have declined, and food imports have become critical for the growing urban areas and some rural communities (Campbell 2015). Traditional foods harvested from the land and sea have, in many instances, been supplanted by cheaper, highly processed, high-sugar imported foods like rice, sugar, soft drinks, and calorie-dense snack foods (Thaman 1988; Campbell 2000; Hawley and McGarvey 2015). In Fiji, a 2015 national nutrition survey found that over 60% of adults were consuming more calories than the recommended daily intake (RDI)—2000 kcal/day—mostly from cereals (rice, flour products including noodles) (Schultz et al. 2019). International trade agreements keep these refined, imported foods cheap, often cheaper than the cost of locally produced foods like cassava and taro (*dalo)* that contain more nutrients (Thow et al. 2015). Furthermore, the availability to purchase these foods in bulk (10 kg), their long shelf life, and the variety of dishes that can be made make these foods more convenient than local foods that need to be harvested daily and spoil in the heat.

Changing nutritional choices at the population and individual levels in PICs have been connected with rising instances of obesity, leading to higher risk for cardiovascular disease, diabetes, and cancer (Hawley and McGarvey 2015). Previous studies in Fiji found incidences of lower diet diversity in rural villages and thus lower nutrient diversity (Medina Hidalgo et al. 2020; O’Meara et al. 2019). Anemia—a lack of iron—affects 40% of children under 5 years and 39% of pregnant women in Fiji, with implications for mental and cognitive development (World Bank 2023). Less than half of the population consume the RDI of calcium and vitamin A, yet these nutrients are found in locally grown green leafy vegetables and fruits (Schultz et al. 2019) and only 15% of Fijians eat the RDI of fruits and vegetables (Ministry of Health and Medical Services 2023). Morgan et al. (2016) found while people liked and understood the importance of consuming fruits and vegetables, their preference for processed and imported foods was increasing. This is especially notable following natural disasters when local food sources can be destroyed, and food aid comes in the form of imported processed foods until new crops can be established.

PICs are exposed to a range of environmental extremes, most notably, tropical cyclones and drought events, under which food security is most tested for many PIC communities.

Climate change is driving the occurrence of more frequent and intense tropical cyclones, altering precipitation regimes, and causing sea levels to rise with implications for agricultural lands and fresh water systems (Nurse et al. 2014). In addition to climate change, human activities are also degrading local ecosystems. Deforestation, some unsustainable farming practices, and resource extraction notably mining are accelerating land erosion and increasing flood risk (Thaman 2002; Wairiu 2017). Meanwhile, urban sprawl is encroaching on agricultural lands and polluting local waterways (Carden 2003; Duncan 2012; McCubbin et al. 2017). The cumulative impacts of these changes are further stressing already stressed food systems in many PIC communities, raising the question “what is the future of food security in PICs under rapidly changing socio-ecological conditions?”.

To date, most studies about food security in PICs have tended to focus on food production at broad country-level scales (Albert et al. 2015; Bell et al. 2009; Iese et al. 2015). Few studies have been conducted at the local food system scale and fewer consider both social and ecological factors that influence food security (e.g. Allen 2015; McCubbin et al. 2017). Local food security studies in Fiji have mostly been conducted in rural villages (Currenti et al. 2019; O’Meara et al. 2019) or on small outer islands (Medina Hidalgo et al. 2020), with less known about the experiences of people living in productive areas on larger islands where there is sufficient land and market access. In this article, our aim is to investigate if households in Narewa village, located in Nadroga-Navosa Province on Viti Levu the largest island and the in most productive agricultural region in Fiji, the Sigatoka Valley known as the “salad bowl of Fiji” (the main producer of fruits and vegetables), are meeting their own food needs and what socio-ecological challenges are impacting their food security. To answer these questions, we characterize the food system in Narewa, document experiences of food security, and discuss socio-ecological factors and processes that influence food system resilience.

## Case study

### Narewa, Nadroga-Navosa, Fiji

The Republic of Fiji lies in the Southwest Pacific Ocean between 177°E–178°W and 16°S–20°S. Of the 332 islands, 100 are inhabited; Viti Levu is the largest island (10,388 km^2^) and where 70% of the 889,953 population live (Central Intelligence Agency (CIA) 2020). The Indigenous inhabitants, *iTaukei*, today make up 56% of the population and own approximately 87% of the land (CIA 2020). Outside of urban centres, *iTaukei* live in villages where life is communal and centers around the village welfare, family, tradition, culture, ritual, and spiritual faiths.

**Fig. 1:**
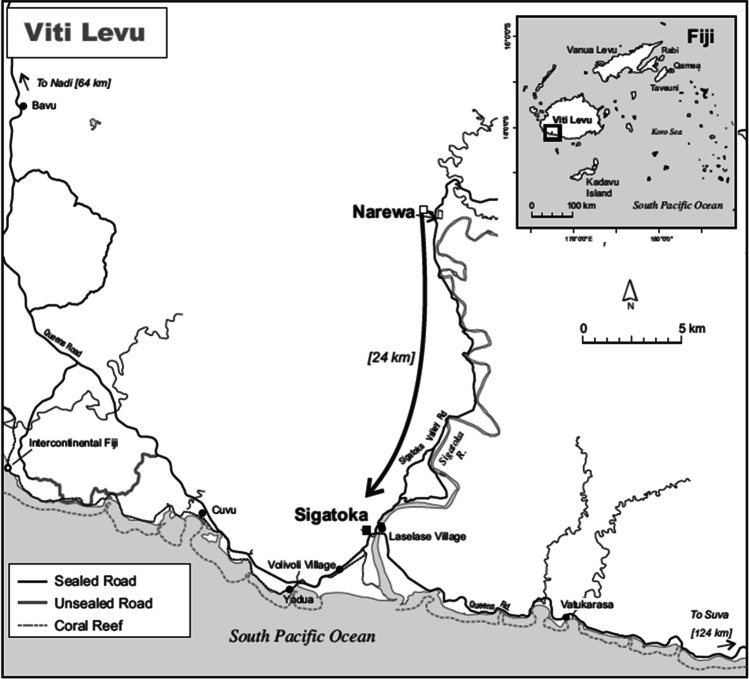
Map showing the location of Narewa and distance to Sigatoka the nearest urban centre

Narewa village is located in Nadroga-Navosa Province the fifth largest of the 15 provinces and is one of 1193 villages in Fiji (Fig. 1). Narewa has a population of 279 people living in 66 houses. A sealed major road provides year-round access to Sigatoka, the nearest town of 10,000 people 24 km away. The entrance to the village is via an 800-m dirt road off the sealed road. Houses are made of concrete blocks, corrugated iron, or bamboo. Most homes access water from one of the communal taps fed by a pipeline from a dam 1 km upstream. Hydroelectricity from the Nadarivatu Dam is available and mostly used for lighting with a third of houses having a refrigerator and fewer an electric oven. Women do most of the cooking either inside on a kerosene stove or over a sheltered open fire beside the house.

The village was originally located on the hilltop to the west of its present location. Due to a dispute over land ownership in 1912, the village was re-settled at the bottom of a valley where people developed farms on the flat land alongside the Naqalitala creek that runs through the present-day village. Before resettlement, the hills were largely forested, but deforestation over the past 50 years, including the planting and logging of pine trees by the government in the 1980s and 1990s, has resulted in soil erosion, increased severity of flooding, and sedimentation in the river. Today, the hills surrounding the valley are covered mostly with low-growing grasses and small pockets of remnant forest. The grasses grow in small clumps, providing food for livestock (goats and cattle) that freely wander the hills. In the dry season, the grasses die, so farmers burn them to generate new green growth to feed the animals. Despite efforts by the Ministry of Agriculture to educate farmers about the harmful effects of these practices on soil erosion, they continue to be prevalent across Fiji.

There are two *matagali* (land-owning clans) in the village (*No-i-Rewadra* and *Nachuwaleka*). *Matagali* farms are less than a 30-min walk from the village and range from flat land to steep hillsides. There is no bridge, so people must walk across the Naqalitala creek to access some of the farms, and this can be difficult during the wet season when the creek is deep. Most farmers monocrop (cassava) or multiple crop (cassava, corn, sweet potato); the farming is physically demanding as the gleysolic soils are hard to work because of their type and topography, and competing native vegetation grows quickly. Many farmers use bullocks to plough and clear the land and those without bullocks must either borrow them or dig with hand tools. Horses and bullocks are used most often to transport crops on wooden sleds, although in the wet season, when the ground is too wet, bamboo rafts are used to float the crops down river to the village. As the soils become waterlogged and unable to grow crops during the wet season, farmers plant on the hilltops and hillsides, which exacerbates soil erosion and slope instability. The dry season is when most of the food is grown. Commonly grown crops include cassava, taro *(dalo)*, sweet potatoes and *bele*, a green leafy vegetable for local consumption, and cucumber as a cash crop. Most families also have fruit trees on their farms, including coconut, banana, plantain, breadfruit, and mango.

## Methods

### Research approach

The research was guided by a community-based participatory research approach that involved working with local agencies, the Nadroga-Navosa Provincial Council (NNPC), and village members as researchers and participants (Balazs and Morello-Frosch 2013; Currenti et al. 2019; Pearce et al. 2021). During initial conversations between the NNPC and researchers, food security and climate change were identified as priority issues. Narewa was identified as a potential case study due to its importance in the province as a food producer and proximity to Sigatoka. The researchers visited the NNPC and Narewa in May 2018 to learn more about food security in Fiji and confirm interest in the research. The researchers returned to Fiji in September 2018 and worked with the NNPC to develop, test, and refine the interview questions with a sample of *iTaukei*. A *sevusevu*^1^ was then conducted in Narewa to communicate the purpose of the research and formally request consent for the research to proceed, with acceptance implying communal consent for all members of the villages to be involved (Nabobo-Baba 2008). The researchers were invited to live in the village for the next month and work with a village member (research assistant) to conduct interviews. An *itatau*^2^ was held the end of the data collection. Study protocols were approved by the Human Research Ethics Committee at the University of the Sunshine Coast (A/15/751). The researchers returned to Narewa in June 2022 to return the research findings. This included a booklet and presentation for the village members and the NNPC. The return was delayed due to travel restriction during the COVID-19 pandemic.

### Data collection

Data were collected using semi-structured interviews (*n* = 25), a fixed question food insecurity experience scale (FIES) (*n* = 25), and a free listing exercise about preferred and consumed foods (*n* = 24). Three types of non-probabilistic sampling were used to recruit participants: purposive sampling was used to recruit participants who were most involved in making food choices in their households, were over 18 years of age, and were permanent residents of Narewa (not visiting from another village); non-quota sampling was used to ensure that participants from vulnerable minorities were included such as single mothers; and snowball sampling involved asking participants and the research assistant to identify people who might be willing to take part in the research. Of the 66 households, 18 women and seven men were surveyed (Table 1). The reason for including more women than men in the sample is that in *iTaukei* culture women are largely responsible for food procurement and preparation.

**Table 1:** Demographic characteristics of participants

Age (years)	Women	Men	Total
18–29	3	1	4
30–39	1	2	3
40–49	5	0	5
50–59	5	2	7
60–69	3	1	4
70–79	1	1	2
Total	18	7	25

Interviews were semi-structured using open-ended and fixed survey questions. Interview questions collected data on basic household demographics, the food system in Narewa, food preferences, and consumption in the past month as well as process and factors that affected food security in the past year (Table 2). Data were collected between September and October 2018, which is the dry season when farms are most productive. Interviews were conducted in a *talanoa* style (face-to-face conversation) as is culturally appropriate with *iTaukei*, in the home of the participant, and in the chosen language of the participant (English or Fijian) (Nabobo-Baba 2008). The researchers were not fluent in Fijian so a research assistant from the village co-led interviews and interpreted between Fijian and English in real-time. After each interview, transcripts were reviewed and verified with the research assistant who was able to interpret key phrases and core meaning.

**Table 2:** Themes and topics covered in the interview guide

Food security pillar	Theme	Topics covered
Availability	Village food system	- Foods produced, harvested, purchased and shared - Relative consumption of local and store foods in the past month
Access	Background information Food security status	- Basic household demographics (age, gender, marital status, household size) - Livelihood activities (occupation; employment status) - Income (includes remittances) - Food security over the past year
Utilization	Food preference	-Food preference and food consumption
Stability	Social and ecological factors affecting food security	- Ecological factors - Social factors - Coping and adaptation strategies

After completing questions about basic household characteristics, participants were asked to complete a free listing task. Free listing is an established ethnography tool used to identify and calculate the cultural salience of items such as food in a cultural domain (Quinlan 2005). Free lists have been used in the PICs to generate cultural salience data on food preference and consumption patterns (Horsey et al. 2019; McCubbin et al. 2017) and adopted for this study. Participants were asked to list all the foods that came to mind when they were asked ‘What foods do you prefer to eat?’ Responses ranged from four to 18 different foods with a median of 10. Free lists in response to ‘What foods do you eat most often?’ ranged from four to 17 food items with a median of eight. All participants took less than 2 min to complete each list. Free listing for cultural salience is based on the assumptions that people list items in order of familiarity and that items listed by most participants indicate locally prominent items.

The FIES was used to assess household food security over the past year. The FIES is a rapid assessment tool consisting of eight yes/no questions that capture behavioural and psychological manifestations of food access insecurity (Ballard et al. 2013) (Table 3). When participants answered yes to any question, they were asked to indicate the frequency of the experience (rarely 1–2 times, sometimes 3–10 times, often 10 + times) and explain why they had become food insecure and how they had reacted. *iTaukei* take pride in their ability to produce food for their family and village hence due to the sensitive nature of the questions, the survey was conducted near the middle of the interview when a level of trust had been built between the participant and researchers.

**Table 3:** Food Insecurity Experience Scale questions (Ballard et al. 2013)

Survey questions		Severity of food insecurity
Preamble: During the past 12 months, was there a time when:		
1	you were worried you would not have enough food to eat because of a lack of money or other resources?	Mild: food quality may be compromised
2	you were unable to eat healthy and nutritious food because of a lack of money or other resources?	
3	you ate only a few kinds of foods because of a lack of money or other resources?	
4	you had to skip a meal because there was not enough money or other resources to get food?	Moderate: food quantity is compromised
5	you ate less than you thought you should because of a lack of money or other resources?	
6	your household ran out of food because of a lack of money or other resources?	
7	you were hungry but did not eat because there was not enough money or other resources for food?	Severe: insufficient food quantity
8	you went without eating for a whole day because of a lack of money or other resources?	

### Data analysis

Data from the semi-structured interviews were analyzed following the principles of latent content analysis to identify recurring or common themes related to the broad categories of food security outlined in the interview guide (Bernard 2012). Data from the fixed questionnaire were entered into a Microsoft Excel spreadsheet and analyzed using descriptive statistics. The food system responses were divided into local and store food for chi-square analysis conducted in XL Stat 2018. Responses to FIES were categorized according to severity as outlined in Ballard et al. (2013). Responses to the FIES were also analyzed alongside household demographic information. The low sample size prevented statistical analysis on most characteristics and on those where it was conducted no statistical variation was detected.

Responses to the free listing questions underwent salience analysis (outlined in Quinlan 2005) to determine which foods were preferred and which foods were eaten most often. Foods were ranked inversely from how the participant listed them and then divided by the rank number of the total number of foods the participant listed. A mean salience score was then calculated for each food item by summing all salience scores for each food and dividing by the number of participants (*n* = 24). A larger salience score indicated greater consensus within the participants. To determine if there was a statistical difference between local and store food preference, a comparison of frequencies to find the mean frequency was conducted. A paired sample *t*-test was done on the mean frequency. The same analysis was applied to the foods eaten most often. To determine if preferred foods were eaten often, a proportional mean was calculated.

### Limitations and future studies

Food security in Fiji is affected by seasonality and location. Data for this study were collected during the end of the dry season when farmed food is plentiful; therefore, caution must be used when generalizing these data over a year. Additionally, Narewa has access to a sealed road giving year-round access to markets unlike those in the remote inland regions or on smaller islands with water barriers that further impact food security. Further studies are needed to be conducted during the wet season when farms are less productive and in a diversity of locations including remote and small islands. Within the context of a village, food sharing was identified as assisting with food security and more research is needed to better understand this cultural practice and how it can be supported.

## Results

### Food availability

All households procured food from family farms, local markets, and the supermarkets in Sigatoka and 84% of households also collected food from the Naqalitala creek or Sigatoka River (Table 4). Eighty percent of households identified having a full-time farmer. Households without a farmer relied upon extended family members to share farm foods with them or they purchase farmed and imported foods at the markets. Sixty percent of households had one member in paid employment, of these 60% were part-time positions filled by women who worked at one of the local farms preparing produce for export and earned approximately USD $9 per day. Some of the cash generated from part-time work was used to purchase food but most was used to pay for other expenses like school supplies, donations to the church, and for special occasions like birthdays and weddings.

**Table 4:** Household procurement and consumption from local and store food source and most frequently consumed foods (*n* = 25)

Measure	Local foods		Store foods
	Farm	River	
Households procuring from each source (%)	100%	84%	100%
Number of unique foods obtained from each source	47	5	52
Average number of foods procured from each source	10	4	12
Foods most frequently procured (% of households)	Cassava 100%	Crab 22%	Flour 100%
	Banana 92%	Eel 22%	Rice 83%
	Orange 50%	Fish 21%	Sugar 83%
	Plantain 50%	Prawns 20%	Tinned fish 75%
	Potato 42%	Mussels 20%	Crackers 71%
	Red bean 42%		Noodles 67%
	Taro 38%		Salt 62%
	Breadfruit 35%		Chicken 54%
	Coconut 35%		Cooking oil 54%
	Yams 35%		Onion 46%
Proportion of food consumed from each source over the past month	48%	18%	34%

Local food availability is affected by seasonality. The most productive time for farming was the dry season when foods such as sweet potatoes, beans, and breadfruit grow compared to the wet season when the waterlogged soils support fewer crops like cassava and taro leaves. In contrast, the village creek was not a source of fish in the dry season as it dried up and people had to walk approximately 2 km to fish in the Sigatoka River. Locally produced foods were the mainstay of most people’s diets (66%). Cassava or sweet potatoes were the dominant food for lunch and supper, and were usually complimented with store-bought rice or flour products^3^ such as roti or scones, and/or fresh fish and other aquatic foods. The purchase of imported store foods is governed by price. Less expensive items like flour, rice, and noodles are considered food staples whereas fresh fish and chicken are considered luxury items and are purchased infrequently.

Several households owned livestock, goats, and cows. These animals were not for household consumption but as a source of income. A goat sold for approximately USD $150 and a cow started at USD $300. It is customary to serve meat such as goat, beef, or pork at special occasions such as funerals and weddings. Pigs were not farmed instead hunters would harvest wild boar when desired for a special occasion. As few people had refrigerators, the meat had to be cooked and consumed within a day.

### Food access

The FIES results show that in the past year most participants self-identified as being food secure (52%) (Table 5). This means that they did not worry about meeting food needs or experience hunger in the past year. Food insecurity was largely experienced as mild (40%). These participants mostly worried about meeting future food needs (36%). Concerns included the potential of flooding and tropical cyclones to damage crops, and not having enough food for special occasions or when visitors came to stay. Some participants indicated that sometimes (3–10 times a year) they were forced to reduce the size of their meals (8%). One participant explained that she was ill for a period of time and her daughter had to stay home to care for her preventing her from working at the export farm, their only source of income. Another participant described how the death of their bullock had reduced the amount of food they could grow and sell, resulting in them sometime reducing their food intake to cassava and tea. No participant reported severe food insecurity causing hunger. Participants explained that they could always eat something from the farm, *kerikeri*^4^ from neighbours, or rely on extended family to share food. “In a Fijian village it is always the same if you have no money, you will not die, if you have no relatives you will die” (Participant 1 27 September 2018).

**Table 5:** Participant responses to the Food Insecurity Experience Scale categorized by severity

Category	Severity	Household (%) (*n* = 25)
Food secure	Food secure	52%
Food insecure	Mild	40%
	Moderate	8%
	Severe	0%

### Food preferences

Free listing sought to identify what foods participants preferred to eat and what foods they were frequently eating. A total of 58 different foods were preferred, with participants listing from four to 18 foods with a median of 10 foods listed per participant. Cassava carried the highest composite salience of 0.91 (Table 6). All participants mentioned cassava and 18 listed it first, signalling it is a preferred food within the village. Taro leaves held the next highest composite salience (0.52). Flour products, including roti, scones, pancakes, and buns, ranked third for composite salience (0.33). Of all the preferred foods listed, 73% were local foods and 27% were store foods. A chi-square test and paired sample *t*-test show a significant preference (DF 24, *t* = 4.3, *p* = 0.000) for local foods supporting this finding. Respondents shared that they preferred local foods because of their freshness, they contained no additives or preservatives, and they liked to be self-sufficient in meeting their own food needs.

**Table 6:** Free listing results of preferred foods listed by seven or more participants

Foods listed by seven or more participants	Salience scores for each food	Composite salience*/n* (*n* = 25)
Cassava	22.75	0.91
Taro leaves	12.91	0.52
Flour products	7.77	0.33
Taro	7.38	0.3
Fish fresh	7.07	0.28
Sweet potato	6.59	0.26
Cabbage English	5.13	0.21
Chicken	4.86	0.19
Moca (Fijian spinach)	4.73	0.18
Bele (leafy green)	4.42	0.17
Tinned fish	3.08	0.12

Cassava was also the food consumed most often (0.72) (Table 7). This was followed by flour products (0.68) and then rice (0.51). With the exception of cassava, other preferred foods were eaten less often. Participants explained that seasonality prevented some preferred foods from being available year-round, access to cash determined if they could purchase preferred foods from the supermarket, and some households focused on growing cash crops such as cucumber and corn rather than preferred foods.

**Table 7:** Free listing results of foods eaten most often by seven or more participants

Foods listed by seven or more participants	Salience score for each food	Composite salience*/n* (*n* = 25)
Cassava	17.88	0.72
Flour products	17.08	0.68
Rice	12.76	0.51
Taro leaves	7.68	0.3
Fish tinned	7.4	0.29
Moca	6.81	0.27
Chicken	6.68	0.26
Noodles	3.34	0.13

### Socio-ecological challenges

Local geography constrains food production in Narewa. Viti Levu has a central mountain range running from north to south and Narewa is situated on the drought-prone leeward side of the range. While annual rainfall averages 2000 mm, it falls mostly in the wet season from November to April, with dry spells at other times of year that can last for 3 to 4 months (Raqona et al. 2020). Much of the rain falls in heavy brief local showers, which causes excessive surface runoff and flows in channels down the multiple steep slopes into lower valleys such as the one Narewa is located in. The surface runoff removes the already shallow fertile topsoil further challenging food production.

Extreme weather events were identified by most participants as having high potential to affect food production (84%). Participants identified that category 5 tropical cyclone Winston in 2016 was the most severe tropical cyclone they had experienced, and that they worried about the impacts of expected future tropical cyclones. Several farmers believed that flooding had intensified since the pine forests on the hills had been logged between 1980 and 1990. The Ministry of Agriculture has encouraged farmers to refrain from burning and to replant the hills with sandalwood trees, which could be harvested in 15-year rotations, but respondents doubted the viability of a sandalwood economy and its effectiveness at combatting soil erosion, calling for longer term solutions.

**Fig. 2:**
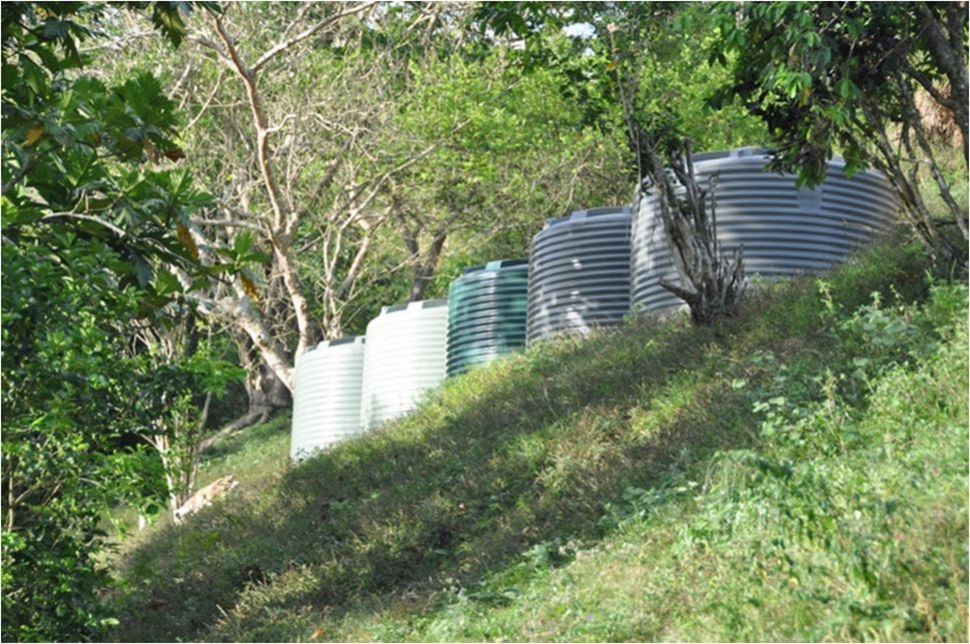
The five water tanks waiting to be connected to the the village infrastructure

Drought was identified as an ongoing challenge for food production. Drought reduced crop size and access to safe drinking water thus undermining food security. During the dry season when water shortages occur, people modified their water use by using pit instead of flush toilets and bathing in stagnant pools in the riverbed. However, modifying water consumption in these ways is insufficient to meet needs, and households with enough income were purchasing bottled water for consumption, an expense that is beyond most households. The Fiji government is aware of the problem and in 2016, as part of the Five-Year, 20-Year Development Plan, it placed 110,000-l water tanks in Narewa to fill weekly by truck with treated water as a safe freshwater source during the drought (Vakasukawaqa 2018). The *turaga-ni-koro* explained the village had developed a strategy to try and solve its freshwater crisis by placing an additional five tanks on a hill in the village (Fig. 2). These tanks would be filled overnight from the village dam and flow to the homes during the day when people needed water. However, at the time of the research, the tanks sat dry as the village needed assistance to connect the tanks to the dam pipeline and the village water infrastructure. The *turaga-ni-koro* explained there had been no follow-up on the water tank program and although the village had requested help they continued to wait. The *turaga-ni-koro* pointed out that the success of the water tank reservoir depends on maintaining the pipeline from the village dam that was old and had multiple breaks and cracks. Furthermore, the increasing water demands from the growing village population for drinking, hygiene, flushing toilets, and washing machines may soon exceed the capacity of the dam.

## Discussion and conclusion

This study examined food security at the household scale in Narewa village, an *iTaukei* village located in the productive Sigatoka Valley in the context of recent socio-ecological changes. The study responds to the expressed need to examine food security in Fiji at the community scale and to consider socio-ecological factors affecting food security (Medina Hidalgo et al. 2020; Mycoo et al. 2022). To this end, data were collected on each pillar of food security, access, availability, and quality using a mixed methods approach. The results show that most households were meeting their preferred food needs through farming, fishing, and local sharing networks. The importance of local food production to household food security is well-recognized in Fiji (Iese et al. 2021; Randin 2020) and in other PIC communities (Allen 2015; Iese et al. 2018; Savage et al. 2020). The finding that people preferred to consume locally produced foods also reinforces what others have found working with villages in Tuvalu (McCubbin et al. 2017) and Samoa (Horsey et al. 2019). However, local food production is exposed and sensitive to seasonal variations, climate change, and societal changes.

The finding that 40% of respondents reported having experienced mild food insecurity in the past year because they worried about the potential impact of extreme weather events like drought, tropical cyclones, and flooding is concerning. Future climate projections show that Fiji will continue to experience an increased frequency and intensity of extreme weather events, which are known to disrupt local food production (Mycoo et al. 2022). In recent years, tropical cyclones have damaged village farms forcing households to purchase less desirable and often nutritionally poor imported foods like flour, rice, and sugar, a trend that has been observed elsewhere in Fiji (Iese et al. 2018). Household incomes, however, are precarious, with most households relying to varying degrees on the sale of farmed cash crops, meaning that if crops are damaged by extreme weather, people also lose an important source of income needed at times when local farm foods are also not readily available. This finding reinforces the importance of local food production for the household economy and the need to make local environments and crop production more climate resilient. For Narewa, having access to fresh water throughout the year is paramount for crop production and human health especially during extreme dry periods, which are projected to worsen in the future. This will require government investment in fresh water infrastructure and the meaningful involvement of village members to ensure that freshwater infrastructure is suitably designed and maintained.

Social cohesion and intra- and inter-community food sharing networks are important for maintaining food security throughout the year and especially at times when the production of some crops is stressed. The results show that farmed foods were most commonly shared when there was a surplus, usually prioritizing elderly- and single female–headed households. During times of stress, households relied on reciprocity from other households with producing farms and/or from family living in other villages. Food sharing as a coping strategy has been documented elsewhere in Fiji (Pearce et al. 2017; Currenti et al. 2019), Tuvalu (McCubbin et al 2015), and in the Federated States of Micronesia, Palau, Papua New Guinea, Solomon Islands, Tonga, and Tuvalu (Ferguson et al. 2022). Sharing depends, however, on the strength of social relationships and assumption that some food production will be spared the impact of an extreme weather event.

The findings of this study show that given that the foundation of food production in Narewa, like other villages in the Sigatoka Valley, relies on the long-term viability of local agricultural systems, focus needs to be placed on the natural resources that form the backbone of these systems (e.g. fresh water availability, soil health, slope stability) and their resilience to anthropogenic and natural stressors. Focusing on nature-based solutions and/or ecosystem-based approaches that restore, maintain, and enhance biodiversity important for food security, such as ecosystem function and services, will also increase the resilience of ecosystems to future climate change impacts. Examples of such efforts include those by the Fiji Ministry of Agriculture and Forestry to promote agroforestry, reforestation, and restoration of riparian zones. The principle of ecosystem-based management should be expanded in Fiji and scaled up to the PIR. Efforts to protect nature should also be combined with greater attention on food storage and the use of resilient crops, knowledge of which may be limited in some villages, including Narewa, requiring some form of outside intervention to share knowledge and facilitate social learning. Efforts that strengthen social cohesion and sharing networks also inadvertently increase people’s capacity to deal with challenges to food security and underpin the resiliency of the village to extreme weather events. Taken together, these efforts, operating across scales—local village to government decision makers—are needed to avoid breaching tipping points in the food systems of Fijian villages at a time of rapid socio-ecological change.

## Data Availability

The data is required to produce the above findings comes from working with an Indigenous Peoples community and cannot be shared at this time due to ethical reasons.

## Footnotes

1 Ceremonial offering of yaqona root to the leadership and community when arriving in a village.

2 Ceremonial offering of yaqona root when a visitor leaves a village.

3 These are flour-based products baked by households unlike noodles which are bought processed and packaged.

4 A relative or neighbour can request something that is needed which is willingly given with no expectation of repayment.
